# Navigating the Complexity of a Bipolar Pregnant Patient With Multiple Comorbidities

**DOI:** 10.7759/cureus.51510

**Published:** 2024-01-02

**Authors:** Paula Traugott, Adriana Medina, Jonathan M Parker

**Affiliations:** 1 Medicine, University of Buenos Aires, Buenos Aires, ARG; 2 Surgery, Ross University School of Medicine, Miramar, USA; 3 Psychiatry, Jackson Behavioral Health Hospital/University of Miami Health System, Miami, USA

**Keywords:** bipolar disorder i, psychogenic nonepileptic seizures, psychiatric management, pregnancy, substance use disorder (sud), epilepsy

## Abstract

Bipolar disorder I (BDI) is a psychiatric disorder characterized by the occurrence of at least one manic episode. Within the scope of neurological disorders, epilepsy and psychogenic nonepileptic seizures (PNES) share clinical features and can be differentiated using electroencephalogram (EEG). Substance use disorder is a condition defined by impaired control, risky use, social impairment, and addictive behaviors. We present the case of a 20-year-old pregnant woman with BDI associated with a history of epilepsy, PNES, and polyvalent substance use. The patient presented to the emergency department via the Baker Act involuntary hold multiple times throughout her pregnancy. Recognizing that the welfare of the mother and child was at risk, the court ordered a two-month commitment of inpatient psychiatric treatment at 30 weeks' gestation to ensure safe delivery. Comprehensive inpatient treatment, including risperidone, levetiracetam, lacosamide, haloperidol, diphenhydramine, lorazepam, and later clozapine, and a discharge plan for both the mother and the child are described in detail. Our goal is to contribute to the growing body of knowledge on the management of complex pregnant patients with psychiatric conditions in order to optimize outcomes for maternal and fetal health.

## Introduction

Bipolar disorder I (BDI) is a mood disorder characterized by at least one manic episode; major depressive episodes are common but not a prerequisite for diagnosis [1]. BDI typically begins at an average age of 18 years old, which may overlap with pregnancy because onset coincides with the reproductive years [1,2]. Adherence to the medication regimen is an essential part of the treatment course, and pregnancy may impose some limitations in this regard. Psychogenic nonepileptic seizures (PNES), also known as pseudoseizures, are categorized as conversion disorders. They are characterized by paroxysmal behavioral, experiential, or motor events that resemble epileptic seizures but without the associated epileptiform electrical brain discharges reflected on electroencephalogram (EEG) [1,3]. Substance use disorder is essentially characterized by a cluster of cognitive, behavioral, and physiological symptoms in an individual who continues to use drugs despite severe substance-related problems [1]. The interactions between psychiatric and neurologic disorders, substance abuse, and pregnancy present a multifaceted challenge for healthcare providers. A comprehensive assessment and treatment plan must be tailored to each patient to ensure the well-being of both the mother and the developing fetus. This case report describes the care and treatment of a pregnant patient with BDI, epilepsy, PNES, and polysubstance abuse to expand and add to the current literature.

## Case presentation

A 20-year-old female patient with a history of BDI, epilepsy, PNES, polyvalent drug use, oppositional defiant disorder, and anxiety presented to the emergency department at Jackson Behavioral Health Hospital in Miami, Florida, via the Baker Act, an involuntary examination ordered by law enforcement requiring a patient to obtain a psychiatric evaluation and allowing the patient to be detained up to 72 hours. This patient was currently pregnant and had a history of multiple involuntary presentations to the hospital via the Baker Act throughout her pregnancy.

Since the beginning of her pregnancy, the patient had been admitted to the inpatient unit several times for aggressive behavior and physical altercations, delusions, hallucinations, paranoia, substance abuse of marijuana and cocaine, self-neglect, living on the streets, concerns for being trafficked, and seizures (due to possible medication nonadherence and/or triggered by cocaine use). Despite a multitude of failed residential psychiatric and substance abuse placements during the first five months of pregnancy, the patient relapsed repeatedly absconding from multiple facilities. Her lack of insight into her condition was also evidenced by her nonadherence to medications and belief that she could manage herself independently at home without the need for re-hospitalization. The patient's obstetric ultrasound, which showed fetal growth restriction, increased concerns for the baby's development. Consequently, the patient and the developing fetus were deemed at risk of both physical harm and medical neglect.

The question that arose at this point was: allow the mother to continue with at-risk behaviors, risking harm to both herself and the baby, or admit her for treatment with less harm to both. Recognizing that the welfare of both the mother and child was at risk, the court ordered a two-month commitment of inpatient psychiatric treatment at 30 weeks' gestation to ensure the safe delivery of the baby.

Psychiatric and neurological inpatient treatment included risperidone, levetiracetam, and lacosamide (LCM). Although the course of treatment showed a promising response after approximately one week, emergency treatment orders (ETOs) with haloperidol and/or diphenhydramine and/or lorazepam had to be ordered throughout the inpatient stay, as needed. At 35 weeks' gestation, clozapine was added to the treatment regime due to the patient's numerous impulsive and unpredictable episodes of aggression toward staff, the nurses, and even herself and concerns for the baby after throwing herself several times onto the floor (pseudoseizure). Clozapine's unique ability to effectively calm severe aggressive patients who are refractory to other treatments influenced this decision (Table 1).

**Table 1:** Psychiatric management of the patient during hospitalization ETOs: emergency treatment orders; mg: milligrams (indicates the medication dosage); PO: per os (indicates that the medications was taken by mouth); IM: intramuscular (indicates that the medication was administered by an injection into a muscle); BID: twice a day (indicates that the medication was taken two times throughout the day); TID: three times a day (indicates that the medication was taken three times throughout the day); Morning: refers to the time at which the medication was taken; Noon: refers to the time at which the medication was taken; Afternoon: refers to the time at which the medication was taken; Bedtime: refers to the time at which the medication was taken

Drug	Risperidone	Levetiracetam	Lacosamide	Haloperidol (ETOs)	Lorazepam (ETOs)	Clozapine
07/02/2023	Negative	Negative	Negative	5 mg IM TID	2 mg IM TID	Negative
07/03/2023	1 mg PO BID	Negative	Negative	5 mg IM in the morning	2 mg IM in the morning	Negative
07/04/2023	1 mg PO in the morning; 2 mg PO at bedtime	Negative	Negative	5 mg IM BID	2 mg IM BID	Negative
07/05/2023-07/06/2023	1 mg PO in the morning; 2 mg at bedtime	1500 mg PO BID	100 mg PO BID	5 mg IM at bedtime	Negative	Negative
07/07/2023-07/08/2023	2 mg PO BID	1500 mg PO BID	100 mg PO BID	5 mg IM at bedtime	Negative	Negative
07/09/2023	2 mg PO BID	1500 mg PO BID	100 mg PO BID	Negative	Negative	Negative
07/10/2023	2 mg PO BID	1500 mg PO BID	100 mg PO BID	5 mg IM in the afternoon	Negative	Negative
07/11/2023	2 mg PO BID	1500 mg PO BID	100 mg PO BID	5 mg IM at noon	Negative	Negative
07/12/2023	2 mg PO BID	1500 mg PO BID	100 mg PO BID	5 mg IM in the afternoon	Negative	Negative
07/13/2023	3 mg PO BID	1500 mg PO BID	100 mg PO BID	Negative	Negative	Negative
07/14/2023	3 mg PO BID	1500 mg PO BID	100 mg PO BID	5 mg IM at bedtime	Negative	Negative
07/15/2023-07/27/2023	3 mg PO BID	1500 mg PO BID	100 mg PO BID	Negative	Negative	Negative
07/28/2023-07/29/2023	3 mg PO BID	1500 mg PO BID	100 mg PO BID	5 mg IM at bedtime	Negative	Negative
07/30/2023-08/03/2023	3 mg PO BID	1500 mg PO BID	100 mg PO BID	Negative	Negative	Negative
08/04/2023	3 mg PO BID	1500 mg PO BID	100 mg PO BID	5 mg IM in the morning	Negative	Negative
08/05/2023-08/17/2023	3 mg PO BID	1500 mg PO BID	100 mg PO BID	Negative	Negative	Negative
08/18/2023	3 mg PO BID	1500 mg PO BID	100 mg PO BID	Negative	Negative	12.5 mg PO in the morning
08/19/2023-08/20/2023	3 mg PO BID	1500 mg PO BID	100 mg PO BID	Negative	Negative	12.5 mg PO BID
08/21/2023	3 mg PO BID	1500 mg PO BID	100 mg PO BID	Negative	Negative	12.5 mg PO in the morning; 25 mg PO at bedtime
08/22/2023	3 mg PO BID	1500 mg PO BID	100 mg PO BID	Negative	Negative	25 mg PO at bedtime
08/23/2023	3 mg PO BID	1500 mg PO BID	100 mg PO BID	Negative	Negative	25 mg BID

The intended course of action for our patient involved induction at 37 weeks of pregnancy with approval of the court. Patients have rights to their own freedoms, and in the state of Florida, the Baker Act permits a 72-hour hold. To extend this period, court filings are made, allowing a court hearing to determine if patients are capable of being safely discharged to the community. The court hearing for our patient permitted a commitment of several months, finding the patient was at high risk of injury to herself, the fetus, and others, through her at-risk behaviors.

Following delivery, the child was assessed by the pediatrics team and deemed appropriate. Upon discharge, the child was placed in the care of the grandmother. Discharge plans involved a new trial of residential placement to help the patient improve insight, achieve longer-term stabilization, and potentially obtain guardianship of her child in the future.

## Discussion

BDI is a severe mood disorder characterized by a course of multiple, recurrent, and remitting episodes that may last months or years. The mean age of onset for the first manic or major depressive episode is approximately 18 years old, which overlaps with the childbearing age of affected women [1,2]. The treatment of BDI in pregnant women is challenging, especially when patients have multiple comorbidities (Table 2).

**Table 2:** Bipolar disorder I diagnostic criteria Adapted from [1]

Criteria	Characteristics
A	Criteria have been met for at least one manic episode
B	The occurrence of the manic and major depressive episode(s) is not better explained by schizoaffective disorder, schizophrenia, schizophreniform disorder, delusional disorder, or other specified or unspecified schizophrenia spectrum and other psychotic disorder

Epilepsy is a neurological condition marked by an inherent susceptibility to seizures, leading to neurobiological, cognitive, psychological, and social implications [4]. PNES, a subtype of conversion disorder, are characterized by paroxysmal episodes of limb shaking that may resemble epileptic seizures [1]. While there is no clinical sign or symptom that definitively distinguishes between the two disorders, certain features have been proposed to aid in differentiation. For instance, fluctuations in consciousness, alternating shaking movements, pelvic thrusting, lateral head shaking, and eye closure observed during the event may suggest PNES. The integration of these clinical observations, along with video-EEG results, plays a crucial role in reaching a precise diagnosis [5].

Substance use disorder is diagnosed based on a pathophysiological pattern of behavior related to the abuse of one or more drugs. All drugs taken in excess have a common pathway in the brain: a direct and intense activation of the reward system, leading to feelings of pleasure and a looming need to consume the drug again. There are several criteria that an individual must meet to be diagnosed with this disorder [1]. In our case report, the two substances abused were marijuana and cocaine (Table 3).

**Table 3:** Cannabis and cocaine use disorder diagnostic criteria Adapted from [1]

A	A problematic pattern of cannabis or cocaine use leading to clinically significant impairment or distress, as manifested by at least two of the following, occurring within a 12-month period:
A1	Cannabis/cocaine is often taken in larger amounts or over a longer period than was intended.
A2	There is a persistent desire or unsuccessful efforts to cut down or control cannabis/cocaine use.
A3	A great deal of time is spent in activities necessary to obtain cannabis/cocaine, use cannabis/cocaine, or recover from its effects.
A4	Craving or a strong desire or urge to use cannabis/cocaine.
A5	Recurrent cannabis/cocaine use resulting in a failure to fulfill major role obligations at work, school, or home.
A6	Continued cannabis/cocaine use despite having persistent or recurrent social or interpersonal problems caused or exacerbated by the effects of cannabis/cocaine.
A7	Important social, occupational, or recreational activities are given up or reduced because of cannabis/cocaine use.
A8	Recurrent cannabis/cocaine use in situations in which it is physically hazardous.
A9	Cannabis/cocaine use is continued despite knowledge of having a persistent or recurrent physical or psychological problem that is likely to have been caused or exacerbated by cannabis/cocaine.
A10	Tolerance, as defined by either of the following: a need for markedly increased amounts of cannabis/cocaine to achieve intoxication or desired effect or markedly diminished effect with continued use of the same amount of cannabis/cocaine.
A11	Withdrawal, as manifested by either of the following: the characteristic withdrawal syndrome for cannabis/cocaine or cannabis/cocaine is taken to relieve or avoid withdrawal symptoms.

When BDI, epilepsy, PNES, and substance use disorders co-occur in a pregnant patient, a difficult scenario arises regarding the best treatment. Our goal is to provide information on the best available treatments, highlighting the drugs taken by our patient. Likewise, we will address the possible adverse effects of these pathologies and their treatments on both the mother and the fetus.

Maternal mental illness and substance abuse during pregnancy have been linked to notable negative perinatal outcomes, such as placental abnormalities, low birth weight, fetal distress, intrauterine growth restriction, preterm birth, neonatal hypoglycemia, and adverse effects on neurological development, among others. Consequently, the potential harm of not treating psychiatric disorders during pregnancy outweighs the risk of potential adverse effects of psychotropic drugs [6,7]. Since pregnant women and neonates are often excluded from scientific studies due to ethical and legal concerns, it is important to clarify that insufficient information is available on optimal dosing regimens, pharmacokinetics, pharmacodynamics, and safety characteristics of drugs during pregnancy, fetus, and neonate [8].

Treatment of BDI in pregnant women requires careful consideration of the risks to the unborn fetus and the risk of relapse of BDI. The greater than 50% risk of relapse of BDI during pregnancy and after birth implies that these medications are often necessary during pregnancy to prevent catastrophic consequences for the mother and child [2,7]. In the case of our patient, the main treatment involved risperidone, although she required ETOs with haloperidol and/or diphenhydramine and/or lorazepam on several occasions. A clozapine titration was begun in the last weeks before delivery.

While all antipsychotics traverse the placenta due to their small size and lipophilic properties, the potential teratogenic effects during pregnancy remain uncertain due to limited studies in pregnant women. Current evidence indicates that the risk of maternal and neonatal adverse events, such as gestational diabetes mellitus, respiratory distress syndrome, and neonatal withdrawal syndrome, is primarily associated with the concomitant use of mood stabilizers [7]. Antipsychotics are Food and Drug Administration (FDA)-approved for treating pregnant patients with bipolar disorder. An estimated 1.3% of pregnancies involve the use of atypical/second-generation antipsychotics, while 0.1% of pregnancies involve the use of typical/first-generation antipsychotics [6].

From the available data, there have been no repeated patterns of anomalies associated with the use of risperidone. It's worth noting that most reported malformations appear to be linked to the concurrent use of other drugs known for their teratogenic effects [7]. When compared to other second-generation antipsychotics, risperidone may be associated with a slightly elevated risk of congenital malformations, although additional research is required to draw more conclusive findings [2].

Regarding the use of haloperidol in pregnancy, most recent reviews showed no increased risk for congenital malformations including limb defects, miscarriages, or stillbirths. However, there are some concerns about the side effects of haloperidol in infants, such as low birth weight [9].

Lorazepam is the most common benzodiazepine (BZD) prescribed during pregnancy, although it is classified by the FDA in risk category D. All BZDs cross the placenta, with the highest rate occurring in the third trimester. Although there is evidence that BZDs can lead to miscarriage, preterm birth, and floppy baby syndrome, a meta-analysis of one million pregnancies has not found increased teratogenic risks. Nevertheless, the evidence is not yet clear, and further studies are needed to better understand the effects of BZDs during pregnancy [10].

Limited studies are available on the use of clozapine in pregnancy despite being labeled category B in pregnancy by the FDA [11]. The evidence to date is partially contradictory: some studies have shown no malformations and minimal complications, while others have shown a low percentage of malformations and some potential neonatal side effects such as low heart rate variability, macrocephaly, neonatal seizures, and floppy baby syndrome [9]. On the other hand, while clozapine has the potential to reduce the seizure threshold in individuals with epilepsy, the associated risk is often contingent on the dosage, ranging from 1% to 6%, particularly during swift titration, an aspect not applicable to our patient's case. Notably, our patient, concurrently receiving two antiepileptic drugs, benefited from an additional layer of protection [11].

The other important comorbidity in our patient was epilepsy. Similar to BDI, the treatment of epilepsy during pregnancy is a difficult scenario because both the epilepsy itself and the antiseizure drugs (ASDs) can have harmful effects on both the mother and child [12]. Seizures pose a danger to pregnant women due to the risk of falls and abdominal trauma, along with the potential for fetal harm from hypoxemia and asphyxia. Although 90% of children born to women with epilepsy are healthy, the condition is associated with an increased fetal risk of prematurity, low birth weight, neonatal hypoglycemia, respiratory distress syndrome, congenital malformations, and other complications. Some evidence suggests that the occurrence of congenital malformations is related to early exposure (first trimester) to ASDs, the use of polytherapy rather than monotherapy, and the dose and type of medication. Currently, approximately 30 ASDs are approved by the FDA [13]. In our patient, epileptic treatment was successfully accomplished by the concomitant use of levetiracetam and LCM. 

Levetiracetam is a second-generation ASD that has the advantages of twice-daily administration, reduced necessity for serum level monitoring, no interaction with other ASDs, and minimal impact on cognitive function [14]. Some studies have shown that patients exposed to levetiracetam have the lowest risk of severe congenital malformations [13].

LCM is a new third-generation ASD used for the treatment of seizure disorders, mostly as add-on therapy. It operates through a special mechanism of action that facilitates the gradual activation of sodium channels, thus preventing the overexcitation of neurons. Accordingly, it has a more favorable side effect profile compared to some older ASDs. Teratogenicity from exposure to LCM during pregnancy is not known, so it has been classified in safety category C. Consequently, despite the increasing use of ASDs, there is still limited and unclear evidence on the effects on the fetus and pregnancy, and further studies should be conducted [15,16].

Neuromodulation therapy, including vagus nerve stimulation, direct-to-brain neurostimulation, and deep brain stimulation, has been proposed for the non-pharmacological treatment of epilepsy during pregnancy. However, there is still insufficient evidence to support the safety of this treatment [13].

There is also an interesting hypothesis in the literature suggesting that the risk of complications associated with drugs used in the treatment of both bipolar disorder and epilepsy could be eliminated by administering these drugs intraventricularly or intrathecally. The pathophysiology underlying this theory is linked to the retention of drugs in the mother's central nervous system, bypassing the blood-brain barrier and, consequently, avoiding harm to the fetus. However, further evidence is required to validate this hypothesis, which holds promise for enhancing the treatment of both conditions and improving maternal and fetal outcomes [17].

The third comorbidity in our patient was the abuse of multiple substances, particularly marijuana and cocaine. The main difference from the previous psychiatric and neurological disorders is that substance abuse poses harmful consequences for both the mother and the fetus and lacks specific treatment. 

Marijuana stands out as the most frequently used substance during pregnancy. Its active ingredient (delta-9-tetrahydrocannabinol) is lipophilic and capable of crossing the placenta, leading to a reduction in fetal folic acid uptake, the deficiency of which has been linked to neural tube defects. In addition to these neurodevelopmental consequences, marijuana use during pregnancy has also been associated with stillbirths and fetal growth retardation. Given the current rise in marijuana use during pregnancy, it is anticipated that further evidence regarding its effects on the fetus and newborn will be gathered in the coming years [18,19].

Cocaine use during pregnancy, along with other stimulants, ranks as the second most commonly used substance after marijuana. Its pathophysiology is linked to increased reuptake of norepinephrine, serotonin, and especially dopamine. Cocaine crosses both the maternal and fetal cerebrovascular barriers, as well as the placenta, resulting in the vasoconstriction of maternal vessels and some possible catastrophic consequences such as utero-placental insufficiency, acidosis, and fetal hypoxia. Other recognized adverse effects of cocaine use during pregnancy encompass prematurity, low birth weight, placental abruption, and perinatal infections [20].

## Conclusions

This case report illustrates the complexity of medical and ethical management. Balancing legal considerations, patient autonomy, and the safety of the mother and unborn child play pivotal roles in determining the course of treatment. While treating BDI in pregnant patients with concomitant diseases presents challenges, our case serves as an example of tailored treatment that can be effective in such complex scenarios. Thus, our report underscores the urgency for new clinical trials involving pregnant women with BDI and other concurrent conditions in order to provide more comprehensive statistical data to contribute to the generation of novel and effective outcomes.
